# Cuproptosis and cuproptosis-related cell death and genes: mechanistic links to spermatogenic cell death

**DOI:** 10.1038/s41420-025-02553-2

**Published:** 2025-06-10

**Authors:** Junjun Li, Nihong Li, Hong Wang, Bin Li, Xinyi Tang, Yanxin Guan, Fang Yang, Guangsen Li, Liang Dong, Renbin Yuan, Xujun Yu

**Affiliations:** 1 Chengdu Fifth People’s Hospital/The Fifth People’s Hospital of Chengdu University of Traditional Chinese Medicine, Chengdu, China; 2 Ziyang Yanjiang People’s Hospital, Ziyang, China; 3 TCM Regulating Metabolic Diseases Key Laboratory of Sichuan Province, Hospital of Chengdu University of Traditional Chinese Medicine, Chengdu, China; 4 School of Medical and Life Sciences, Chengdu University of Traditional Chinese Medicine, Chengdu, China

**Keywords:** Infertility, Cell death

## Abstract

Spermatogenesis, which is regulated by multiple cell death mechanisms, is an extremely complex process. The significance of cell death during spermatogenesis is a topic of interest because of its potential medical implications. Cuproptosis is a new mechanism of cell death discovered in recent years, and recent studies have preliminarily confirmed that cuproptosis is involved in the process of spermatogenic cell death, but its specific role in the process of spermatogenic cell death is still unclear. In this review, the mechanisms of spermatogenic cell death associated with cuproptosis and the effects of key genes of cuproptosis on spermatogenesis are discussed together with some new perspectives for the study of spermatogenic cell death.

## Facts

1. Cuproptosis is a novel form of cell death that exists in spermatogenic cells, and many cuproptosis related genes are associated with dysfunction in spermatogenesis.
2. The regulatory mechanism of cuproptosis in spermatogenic cells is still unclear, and there may be a potential connection between cuproptosis and other types of cell death.
3. In the future, in-depth research and understanding of the relationship between cuproptosis and other types of spermatogenic cell death is beneficial for a more thorough understanding of the role of cuproptosis in the mechanisms of spermatogenic cell death.

## Introduction

Spermatogenesis is in a dynamic balance between cell proliferation and death. Several different types of cell death, which are commonly present in germ cells during spermatogenesis and maturation, are of great significance in eliminating abnormal germ cells and provide conditions for accurate transmission of genetic information. However, the increase in germ cells death can lead to a decrease in quality and quantity of sperm, causing male infertility. Multiple factors can lead to an increase in the mortality rate of spermatogenic cells. Intrinsic factors mainly include lifestyle factors and obesity [1, 2]. External factors mainly include physical factors (such as high temperature [3]), radiation [4], chemical factors (such as nitrite [5]), environmental pollutants(such as di-(2-ethylhexyl) phthalate [6]), and biological factors (such as Zearalenone [7]). In recent years, research on cell death mechanisms has gradually deepened, and the known ways of germ cells death mainly include ferroptosis, apoptosis, pyroptosis, and autophagy, etc. These cell death modes each have different morphological and biochemical characteristics. However, there are still many unknown fields regarding the mechanism of cell death. Further exploration of the scope of cell death is beneficial for a more thorough understanding of spermatogenic cell death.

In 2022, Tsvetkov et al. [8] reported a new way of cell death based on mitochondrial respiration and the tricarboxylic acid cycle (TCA cycle), which is named cuproptosis. Cuproptosis is separate from existing other types of cell death, the accumulation of Cu^2+^ in cells is the fundamental condition for cuproptosis. When Cu^2+^ amasses in cells that rely on mitochondrial respiration, excess Cu^2+^ within cells can be transported to the mitochondria by ionophores, and ferridoxin 1 (FDX1) reduces Cu^2+^ to Cu^+^. Cu^+^ combined with lipoylated drolipoamide S-acetyltransferase (DLAT), inducing heteropolymerization of DLAT. The increase in insoluble DLAT leads to cytotoxicity and induces cell death [9]. Previous studies have shown that copper exposure can cause testicular damage and death of reproductive cells [10, 11]. Mitochondrial respiration dependent cells are more prone to copper death [8]. Testicular germ cells rely on mitochondrial respiration, and mitochondria play important roles in various stages of sperm development, maturation, motility, and fertilization. Multiple studies have shown that the impairment of spermatogenic function is related to TCA cycle and mitochondrial function [12–14], which are key links to cuproptosis. Our previous research also showed that copper induction can lead to cuproptosis in mouse testicular germ cells [15], which suggests that cuproptosis may be one of the important mechanisms leading to germ cell death.

## Relationship between cuproptosis and impaired spermatogenic function

**Fig. 1:**
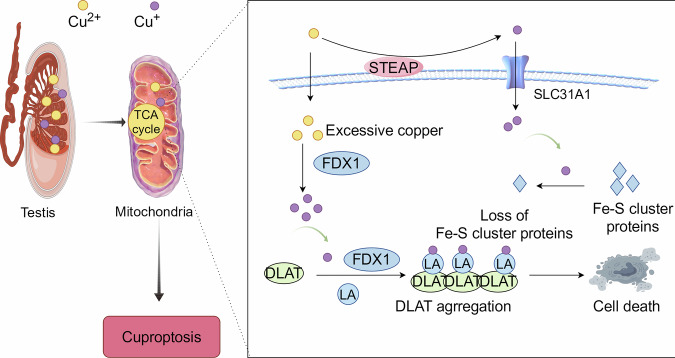
Molecular mechanisms of cuproptosis in the testis. Excess Cu^2+^ within cells can be transported to the mitochondria by ionophores, and ferridoxin 1 (FDX1) reduces Cu^2+^ to Cu^+^. The binding of copper to lipoylated TCA cycle protein leads to lipoylated oligomerization of DLAT, which promotes the aggregation of lipoylated proteins and the loss of the Fe-S cluster proteins.

In cuproptosis, FDX1 is a fundamental regulatory factor, and excessive copper ions in cells can induce an increase in the expression level of FDX1, resulting in protein lipoylated. The binding of copper to lipoylated TCA cycle protein leads to lipoylated oligomerization of DLAT, which makes the aggregation of lipoylated proteins and the instability of Fe-S cluster proteins [8], bringing protein toxicity stress response and ultimately cell death. Our previous study [15] has shown that copper homeostasis is disrupted in the testes of copper-overloaded mice, and the expression of FDX1 is significantly upregulated with a significant increase in testicular tissue cell apoptosis rate, suggesting that cuproptosis may be involved in the death of spermatogenic cells. In addition, the decreased ATP levels and mitochondrial dysfunction were observed, further confirming the mitochondrial damage caused by cuproptosis. In further analysis, pathological lesions and disruption of the blood-testis barrier were found in testes of copper overloaded mice, accompanied by a decrease in sperm’s number and vitality. These research evidence suggests a connection between cuproptosis and impaired spermatogenic function (Fig. 1). Next, we described the potential impact of the ways of spermatogenic cell death associated with cuproptosis and cuproptosis key genes on spermatogenic function. Excess Cu^2+^ within cells can be transported to the mitochondria by ionophores, and FDX1 reduces Cu^2+^ to Cu^+^. The binding of copper to lipoylated TCA cycle protein leads to lipoylated oligomerization of DLAT, which promotes the aggregation of lipoylated proteins and the loss of the Fe-S cluster.

## Spermatogenic cell death associated with cuproptosis

As the basic condition for cuproptosis, copper is required for the activity of various metalloenzymes involved in energy and antioxidant metabolism [16]. Copper also contributes to maintain the function of the cellular redox system, thereby protecting spermatogenic cells from oxidative damage [17]. Copper catalyzes the Fenton-like reaction and generates reactive oxygen species (ROS) and hydroxyl radicals via the Fenton and Haber-Weiss reactions. ROS have a biphasic effect, with appropriate levels of ROS having physiological effects on spermatogenesis, while excessive ROS can lead to oxidative damage in the reproductive microenvironment. Copper exposure causes oxidative stress accompanied by increased ROS and MDA in the testis, leading to decreased sperm quality and structural abnormalities in the testis, germ cell apoptosis, and vacuolization of the Sertoli cells [18]. Meanwhile, increased levels of ROS trigger DNA instability and lipid peroxidation in spermatozoa [19]. Moreover, disruptions in copper metabolism during cuproptosis lead to copper accumulation. Excessive copper can trigger oxidative stress, thereby accelerating apoptosis and autophagy in testicular cells [20]. Subsequently, the p53 signaling pathway was activated in apoptosis and autophagy, enhancing FDX1 synthesis and accelerating the occurrence of cuproptosis [21]. Accumulated copper leads to the buildup of ROS and lipid peroxides while simultaneously depleting Glutathione (GSH), promoting both ferroptosis and cuproptosis [22]. In addition, copper exposure increases the expression caspase-1 and pyroptosis-related genes, such as interleukin-1B (IL1B) and NLR family pyrin domain containing 3 (NLRP3), leading to the induction of pyroptosis [23]. These research evidences suggest a potential connection between cuproptosis and other types of cell death (autophagy, apoptosis, pyroptosis, and ferroptosis). Due to the complex mechanisms of cell death, a deeper understanding of the relationship between cuproptosis and other types of spermatogenic cell death is beneficial for a more thorough understanding of the role of cuproptosis in the mechanisms of spermatogenic cell death. Next, we mainly described other types of spermatogenic cell death associated with cuproptosis.

### Ferroptosis associated with cuproptosis

**Fig. 2:**
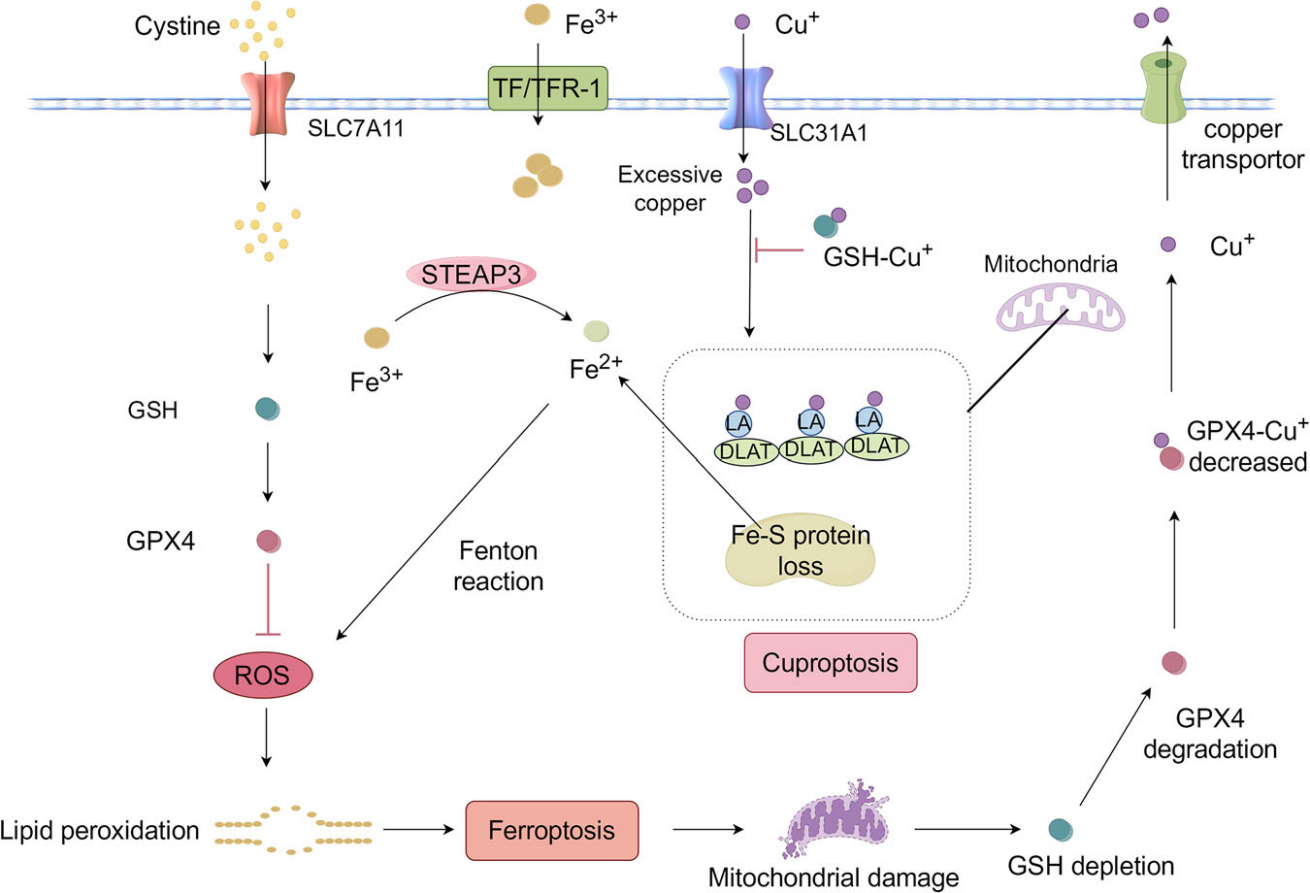
Potential relationship between cuproptosis and ferroptosis. GSH plays an important role in ferroptosis and cuproptosis. Excessive Fe^2+^ produces ROS through the Fenton reaction, which causes lipid peroxidation and ferroptosis. GSH can clear the excess ROS and inhibit ferroptosis. GSH regulates Cu^+^ concentration and can reduce Cu^+^ concentration by promoting intracellular Cu+ transport. In addition, GSH binds to Cu^+^ and can effectively inhibit DLAT aggregation and Fe-S protein loss, thereby inhibiting cuproptosis. Ferroptosis damages mitochondria, further depleting GSH. When GSH is reduced, GXP4 is also reduced and intracellular copper regulation is inhibited, leading to cuproptosis. At the same time, Fe-S cluster proteins are lost, producing more iron ions and aggravating ferroptosis.

Previous studies have shown that the number of cuproptosis regulatory factors is closely related to the number of ferroptosis regulatory factors in different tumor cells, and knocking down ferroptosis key proteins in cells results in significant changes in cuproptosis [24]. Moreover, both ferroptosis and cuproptosis are closely related to mitochondrial metabolism, indicating a significant correlation between ferroptosis and cuproptosis [8, 25]. In the testes, copper sulfate not only induces mitochondrial toxic stress leading to cuproptosis [15], but also drives ferroptosis by affecting the degradation of Glutathione Peroxidase 4 (GPX4) [26], suggesting a close relationship between ferroptosis and cuproptosis in the testes. GSH, as an important intracellular regulator of metabolites, may be a common key link in regulating ferroptosis and cuproptosis [27] (Fig. 2). On the one hand, GSH acts as a substrate for GPX4 to reduce lipid peroxidation and alleviate ferroptosis [28]. On the other hand, GSH controls the concentration of susceptible copper ion pools, reduces the aggregation of lipoylated proteins, and inhibits cuproposis [8]. GSH exhibits inhibitory effects on both ferroptosis and cuproposis, and the inhibition of GSH can induce both ferroptosis [29] and cuproposis [8]. When GSH is inhibited, the elimination of ROS is weakened, and excess Fe^2+^ produces more ROS through the Fenton reaction, which eventually leads to ferroptosis. At the same time, the damage of mitochondria aggravates the reduction of GSH, which weakens the intracellular Cu^+^ clearance. The excess Cu^+^ occurs cuproposis through the aggregation of lipoylated proteins and the loss of Fe-S proteins. In addition, more Fe^2+^ is released to increase intracellular oxidative stress, forming a ferroptosis-cuproptosis self-cycle [30]. Thus, GSH may be a common key link between cuproptosis and ferroptosis.

### Apoptosis associated with cuproptosis

**Fig. 3:**
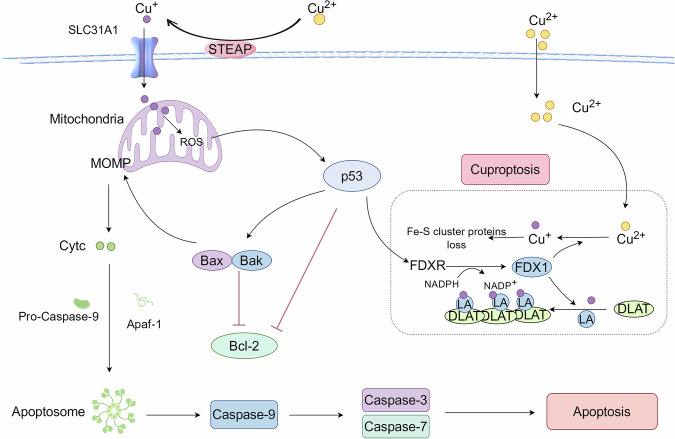
Potential relationship between cuproptosis and apoptosis. p53 may be a key link between cuproptosis and apoptosis. p53 regulates the expression of FDXR, which encodes a ferredoxin reductase responsible for transferring electrons from NADPH to FDX1, thus realizing Fe-S cluster biogenesis. FDX1 converts Cu^2+^ to Cu^+^ and promotes DlTA protein aggregation and the loss of Fe-S cluster proteins. Copper exposure induces oxidative stress, which is accompanied by elevated levels of ROS, thus inducing cuproptosis. Excess copper leads to the production of excess ROS, which results in p53 activation and subsequent activation of pro-apoptotic BH3 members of the Bcl-2 family (Bax, Bak). Bax and Bak neutralize the antiapoptotic protein Bcl-2, disrupting mitochondrial outer membrane permeabilization (MOMP) so that mitochondrial proteins spread into the cytosol. Cytochrome c (Cytc) binds and activates Apaf-1 and pro-caspase-9 to form apoptosomes. In the apoptosome, caspase-9 is activated by autoproteolytic cleavage, initiating a caspase cascade reaction that leads to programmed apoptosis.

A key feature in copper-induced testicular cuproptosis is copper metabolism disorder, leading to copper overload [15]. Copper overload leads to changes in mitochondrial membrane potential, triggering the release of cytochrome C and the generation of excessive ROS. Ultimately, these changes activate the Caspase-9/3/7 signaling pathway, resulting in testicular apoptosis [31, 32]. Excessive copper ions trigger concurrent cuproptosis and apoptosis, indicating a potential relationship between cuproptosis and apoptosis in the testis (Fig. 3). Copper exposure can induce excessive ROS production to damage DNA, triggering the p53 signaling pathway in apoptosis and cell cycle arrest [33]. P53 may be a key link between apoptosis and cuproptosis. On the one hand, p53 regulates the expression of multiple genes involved in Fe-S cluster biogenesis. One of these genes is FDXR, which encodes a ferritin reductase. FDXR is responsible for transferring electrons from NADPH to FDX1, and then to cytochrome P450. This process is key to achieving Fe-S cluster protein biogenesis [21]. FDX1 is a key protein in cuproptosis, reducing non-toxic Cu^2+^ to toxic Cu^+^. Excessive accumulation of Cu^+^ leads to the aggregation of lipoylated proteins and loss of Fe-S cluster proteins, resulting in protein toxicity and cell death [34]. On the other hand, p53 plays a crucial role in mitochondrial apoptosis. Activation of p53 promotes the expression of pro-apoptotic proteins Bax and Bak, inhibits the expression of anti-apoptotic protein Bcl-2. In addition, p53 activates apoptosis-related factors and induces cell apoptosis [35]. Therefore, there is a significant correlation between cuproptosis and apoptosis, and p53 may play an important bridging role in their association.

### Autophagy associated with cuproptosis

**Fig. 4:**
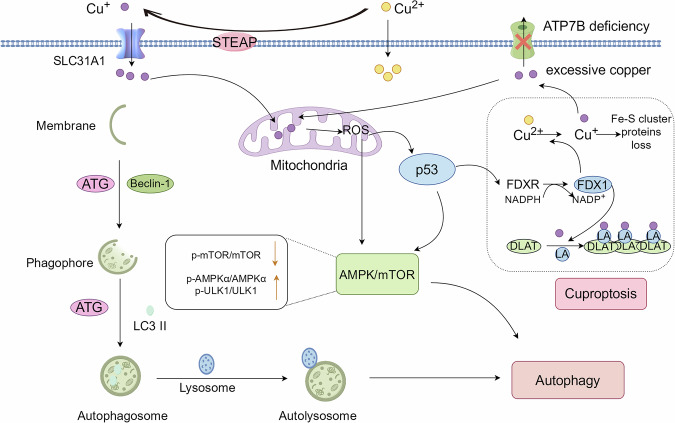
Potential relationship between cuproptosis and autophagy. p53 and ATP7B are the critical link between cuproptosis and autophagy. P53 not only promotes cuproptosis by regulating FDXR gene expression but also promotes autophagy by activating the AMPK/mTOR signaling pathway. Suppression of ATP7B disrupts Cu+ efflux, resulting in progressive copper accumulation that induces cuproptosis and autophagy.

It has been shown that CuSO_4_ induces autophagy in mouse GC-1 cells and testis through the oxidative stress-dependent AMPK/mTOR pathway by downregulating p-mTOR/mTOR and subsequently upregulating p-AMPKα/AMPKα and p-ULK1/ULK1 [26]. In mouse testicular tissues, CuSO_4_-mediated induction of both autophagy and cuproptosis [15] reveals a potential connection between autophagy and cuproptosis (Fig. 4). Copper induces oxidative stress by catalyzing ROS generation via Fenton and Haber-Weiss reactions, thereby activating p53 [36]. p53 serves as a redox-sensitive hub that coordinates oxidative stress in apoptosis and autophagy. Thus, it may play an important role in the link between autophagy and cuproptosis. On the one hand, p53 affects the biogenesis of Fe-S cluster proteins by regulating the expression of the FDXR gene, leading to a deficiency of Fe-S clusters and ultimately inducing cuproptosis [21]. On the other hand, p53 promotes autophagy by transactivating its target genes, mainly by activating the p53/AMPK/mTOR signaling pathway. The AMPK/mTOR signaling pathway is an important regulatory pathway for cellular autophagy, and oxidative stress can activate the AMPK/mTOR signaling pathway [37]. Additionally, ATPase Cu-Transporting 7 Beta (ATP7B) is a critical copper transporter whose downregulation has been demonstrated to disrupt copper metabolism, resulting in intracellular copper accumulation and the triggering of cuproptosis [38]. ATP7B deficiency promotes transcription factor EB nuclear translocation through reduced mTOR activity, thereby significantly upregulating autophagy-related gene expression [39]. In summary, cuproptosis and autophagy are closely related, and P53 and ATP7B may be critical in the association between cuproptosis and autophagy.

### Pyroptosis associated with cuproptosis

**Fig. 5:**
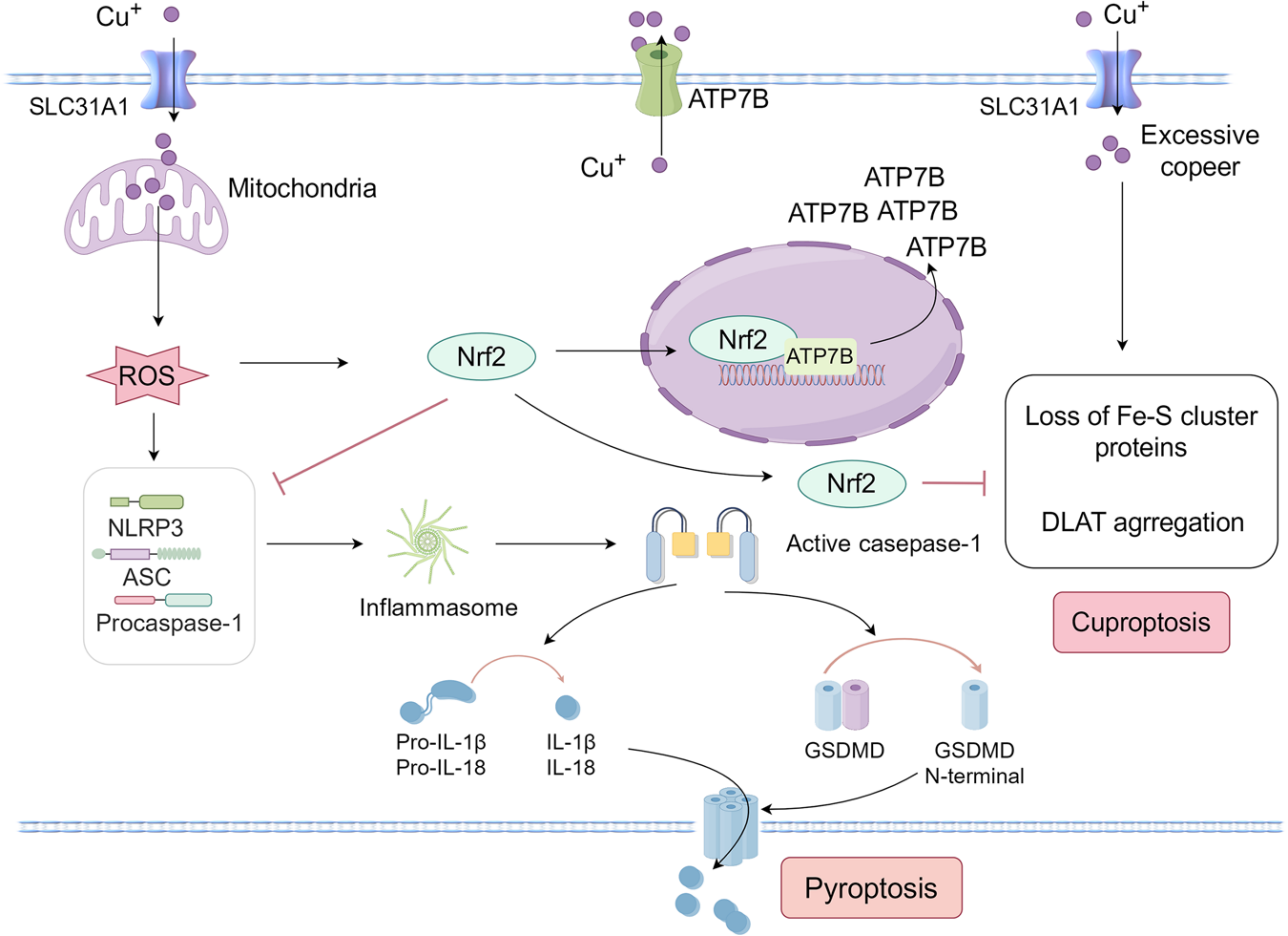
Potential relationship between cuproptosis and pyroptosis. Nrf2 may be an important hub for cuproptosis and pyroptosis. Copper catalyzes the Fenton-like reaction and generates ROS, leading to cellular pyroptosis. Nucleotide oligomerization structural domain-like receptor protein 3 (NLRP3) binds to an apoptosis-associated speck-like protein (ASC), which recruits the pro-caspase-1 to assemble into the NLRP3 inflammasome. Activated caspase-1 cleaves the GSDMD protein to form GSDMD with an N-terminal sequence, which translocates to bind to the cell membrane to form a membrane pore. This process leads to cell death and the secretion of pro-inflammatory mediators, including interleukin 18 (IL-18) and interleukin-1β (IL-1β). However, excessive ROS activate Nrf2, inhibit the production of inflammasomes, and inhibit pyroptosis. In addition, Nrf2 stimulates the synthesis of ATP7B to alleviate cuproptosis by transporting excess intracellular copper.

Pyroptosis can affect testicular spermatogenesis. Excessive ROS can induce damage to the testes and spermatogenic cells, promoting the expression of caspase-1, interleukin-1β (IL-1β), and NLRP3, thereby triggering pyroptosis [6, 40]. Excessive copper leads to the accumulation of ROS in cells [41], and ROS is considered a key factor in activating pyroptosis [40]. Therefore, copper exposure may induce pyroptosis by generating excessive ROS. Copper accumulation is a hallmark feature of cuproptosis, there may be a potential connection between cuproptosis and pyroptosis (Fig. 5). Activation of nuclear factor erythroid 2-related factor 2 (Nrf2) is a member of the transcription factor family, playing a key role in regulating cellular redox homeostasis by inducing various detoxifying and antioxidant enzymes. Elevated ROS levels trigger Nrf2 signaling pathway activation for ROS elimination and tissue homeostatic maintenance [42]. Nrf2 inhibits the production of NLRP3 inflammasomes and prevents pyroptosis [43]. Meanwhile, Nrf2 binds to the ATP7B promoter, enhancing the expression of ATP7B protein and transferring excess Cu^+^ from the cell to the extracellular space, thereby reducing cuproptosis [44]. To summarise, there is a potential correlation between cuproptosis and pyroptosis. The Nrf2 may be a central mediator in the association between cuproptosis and pyroptosis.

## The association between cuproptosis key genes and impaired spermatogenic function

Ten key genes were dentified for cuproptosis, including positive regulation factors FDX1, DLAT, lipoyltransferase 1 (LIPT1), pyruvate dehydrogenase E1 subunit alpha 1 (PDHA1), lipoic acid synthetase (LIAS), pyruvate dehydrogenase E1 subunit beta, glutaminase (GLS), dihydrolipoamide dehydrogenase (DLD), cyclin-dependent kinase inhibitor 2A (CDKN2A), and negative regulatory factor metal-regulatory transcription factor-1 (MTF1). Several cuproptosis genes may be involved in spermatogenic function, and the following are the effects of their related genes on spermatogenesis (Table 1).

**Table 1:** The function of cuproptosis-related genes in spermatogenesis.

Gene	Function
FDX1	FDX1 reduces mitochondrial respiration and affects metabolism, which in turn affects spermatogenesis. FDX1 also affects spermatogenesis by impairing the function, decreasing the number of supporting cells and mesenchymal stromal cells, and by degenerating and necrotizing the vacuoles of spermatogenic cells.
GLS	GLS maintains spermatogenesis by controlling cellular redox homeostasis, eliminating excess ROS, and avoiding spermatocyte oxidative stress-induced apoptosis.
PDHA1	PDHA1 causes spermatogenic disorders by promoting CD4 + T cell infiltration and mitochondrial apoptosis pathways.
LIAS	LIAS provides energy for spermatogenesis by maintaining the activity of the PDH complex through lipoic acid. Lipoic acid avoids the accumulation of ROS, reduces oxidative stress in sperm cells and damage to the testicular microenvironment.
DLD	DLD may promote ROS generation and cause DNA damage, and promote apoptosis of spermatogenic cells.
MTF1	MTF1 inhibits the mitochondrial apoptosis pathway and maintains sperm viability and DNA integrity. MTF1 reduces double-stranded DNA breaks and S-phase arrest, inhibits cell death due to ROS production, and maintains spermatogenesis.
CDKN2A	CDKN2A causes cell cycle arrest in the G1 phase and cellular senescence, affecting spermatogenesis.
SLC31A1/CTR1	High expression of CTR1 specificity in the testis is essential for spermatogenesis.
DLAT	DLAT may be involved in the pathogenesis of spermatogenic disorders through metabolic regulation, oxidative stress, autophagy, and immune microenvironment.
DBT	It may affect the apoptosis of spermatogenic cells by adjusting the Bcl-2/Bax ratio and changing the stability of mitochondrial membranes.
ATP7A/ATP7B	Maintain the microenvironment required for spermatogenesis by precisely regulating copper homeostasis.
GCSH	May affect spermatogenesis by affecting the JAK-STAT signaling pathway.

### FDX1

Mammalian mitochondria contain several ferredoxin proteins, among which FDX1 plays a major role in mitochondrial respiration and energy metabolism, and is a key gene in the regulation of cuproptosis. In cells with knockdown of FDX1, copper, and heme a/a3 levels are decreased, affecting cytochrome c oxidase and NADH production, which in turn reduces mitochondrial respiration [45]. In addition, FDX1-deficient cells produce less ATP, along with abnormalities in fatty acid oxidation, amino acid metabolism, and glucose metabolism, which may lead to abnormal spermatogenesis [46]. FDX1 is present in Leydig cells, Sertoli cells, and spermatogenic cells during testicular development, and there is a significant increase in FDX1 positivity after the occurrence of cuproptosis, along with Sertoli cells, Leydig cells decreased and spermatogenic cell vacuoles degenerated and necrotic [15]. It has been shown that deficiency of FDX1b, the paralogous homolog encoded by FDX1, downregulates the expression of insulin-like peptide 3 (INSL3), insulin-like growth factor 3 (IGF3), and inhibin subunit a (INHA), whereas INSL3 and IGF3 are involved in the differentiation and proliferation of spermatogenesis A spermatogonia, and inha is used for the regulation of FSH, which has a significant role in Sertoli cell proliferation and function, and when their expression is reduced, spermatogonia proliferation and differentiation are abnormal and the function of the Sertoli cells is impaired, which in turn affects spermatogenesis [47, 48]. In conclusion, FDX1 may affect spermatogenesis by influencing metabolism or spermatogonia proliferation and differentiation.

### GLS

GLS is an enzyme mainly located in mitochondria that catalyzes the hydrolysis of glutamine to glutamate. GLS is classified into two subtypes GLS1 and GLS2 [49]. GLS1 is associated with cellular senescence [50]. In the recent study, it was reported [51] that GLS2 gene activity is essential for nematode sperm function and maintains sperm function by controlling cellular redox homeostasis. The tumor suppressor gene P53, expressed in mammalian primary spermatocytes, plays an important role in spermatogenesis during meiotic prophase. This role of P53 may be related to its up-regulation of the function of many antioxidant genes, including GLS2, which acts as a target gene for the tumor suppressor P53 and mediates the function of the P53 protein in regulating cellular energy metabolism and antioxidant defense mechanisms [52]. GLS2 regulates cellular energy metabolism by increasing glutamine hydrolysis, which increases mitochondrial respiration rate and ATP production, it also further increases reduced glutathione, an important antioxidant molecule, and ROS scavenger, by increasing glutamate, which reduces the level of ROS and enhances cellular antioxidant defenses, allowing cells to avoid oxidative stress-induced apoptosis [53]. Thus GLS maintains spermatogenesis function by reducing oxidative stress through antioxidants.

### PDHA1

PDHA1 is one of the proteins involved in aerobic glycolysis. Cuproptosis-related molecular Cluster 2 in spermatogenic dysfunction had high PDHA1 expression, and a large number of CD4^+^ T cell infiltrates [54], whereas in a model of autoimmune testicular inflammation characterized by spermatogenic dysfunction established, the spermatogonial tubules are predominantly infiltrated by CD4^+^ T cell, further suggesting a correlation between cuproptosis and immune infiltration of the testis [55]. PDHA1 overexpression promotes mitochondrial respiration, leading to excessive ROS production and apoptosis [56]. PDHA1 overexpression activates the mitochondrial pathway of apoptosis, and the ratio of pro-apoptotic protein Bax to anti apoptotic protein Bcl-2 increases, leading to the loss of mitochondrial membrane potential and the release of cytochrome C from mitochondria into the cytoplasm, promotes apoptotic body formation, and activates the caspase, and then execute apoptosis [57]. Therefore, PDHA1 causes spermatogenic disorders by promoting CD4^+^ T cell infiltration and mitochondrial apoptosis pathway.

### LIAS

LIAS is a key enzyme in mitochondria, and its catalytic synthesis of lipoic acid plays an important role in mitochondrial energy metabolism and antioxidant defense [58]. Lipoic acid affects energy metabolism by maintaining the activity of the PDH complex in the TCA. Studies have shown that lipoic acid has a positive effect on oxidative stress caused by metals in the body, which can chelate these metal ions, eliminate excess ROS, and facilitate the regeneration of glutathione, vitamin C, and vitamin E [59]. The downregulation of LIAS in spermatogenesis disorders patients leads to a decrease in lipoic acid, which affects the function of the PDH complex, thereby inhibiting TCA circulation and energy supply, which may lead to spermatogenesis arrest (such as meiosis disorder), while lipoic acid deficiency leads to ROS accumulation, damages ROS-sensitive sperm cells, promotes resting memory CD4 T cell infiltration, triggers local inflammatory response, destroys the integrity of the blood-testicular barrier, and aggravates testicular microenvironment damage [54].

### DLD

DLD is a mitochondrial enzyme belonging to the pyruvate dehydrogenase complex, which is one of the key genes for cupproposis [60]. The important role of DLD in cell death has been suggested, DLD can produce large amounts of ROS in melanoma cells, leading to apoptosis [61]. Similarly, during spermatogenesis, DLD produces ROS in redox reactions, and a small amount of ROS is involved in sperm acrosome reaction, capacitation, hyperactivation, and sperm-oocyte interaction, but excessive ROS production can cause oxidative stress, leading to sperm DNA damage and promoting spermatogenic cell apoptosis [62].

### MTF1

MTF1 encodes a metal transcription factor that activates metallothionein to protect cells from heavy metals such as cadmium, zinc, and copper. MTF1 was positively correlated with TIMP2 (metalloproteinase tissue inhibitor), which has the effect of maintaining sperm membrane integrity and preventing sperm DNA fragmentation [63]. In Cd exposure models, increased MTF1 expression inhibited mitochondrial apoptosis pathways (e.g., decreased Bax/Caspase-3, increased Bcl-2) and maintained sperm viability and DNA integrity [64]. Previous studies have inferred that MTF1 overexpression can reduce double-stranded DNA fragmentation and S phase arrest in spermatogenic cells, while inhibiting MTF1 expression leads to inhibition of spermatogenic cells and support cell proliferation, increased ROS production, and then promotes spermatogenic cell death [65, 66].

### CDKN2A

CDKN2A is a gene encoding the P16 protein, which binds to CDK4 and CDK6 and inhibits the formation of kinase-active complexes between CDK4 and cyclin D, thereby regulating the cell cycle and arresting cells in the G phase [67, 68]. CDKN2A mainly acts on the G1 phase and blocks CDK activity, reducing cellular senescence and cell cycle arrest by inhibiting CDKN2A [69]. During spermatogenesis, if CDKN2A is overexpressed, it may arrest the development of spermatogenic cells in the G1 phase or promote cell senescence and finally lead to abnormal spermatogenesis.

### SLC31A1/CTR1

Aberrant expression of SLC31A1/CTR1, a transmembrane protein that plays an important role in copper homeostasis and cellular copper uptake, leads to copper accumulation, which in turn triggers cuproposis [70]. Mice with high expression of CTR1 in the testes and specific knockout of CTR1 began to lose germ cells at the 28th day of life and had hypoplasia in the testis, suggesting that CTR1 is essential for spermatogenesis [71]. It has been shown that oxidative stress promotes the transcription of CTR1 by upregulating specific protein 1 (SP1), thereby increasing cellular copper uptake and FDX1 expression and causing cuproposis [72]. thereby, oxidative stress may also lead to abnormal spermatogenic cell death and ultimately to abnormal spermatogenesis by regulating CRT1.

### DLAT

DLAT is a key regulatory gene for cuproptosis, and as a core component of pyruvate dehydrogenase complex (PDC), it is involved in mitochondrial respiration and TCA cycle metabolism, affecting ATP production [73]. Spermatogenesis is dependent on mitochondria [74], abnormal expression of DLAT not only causes disruption of the TCA cycle [75] and insufficient energy supply to spermatogenic cells, affecting spermatogenesis, but also aggravates oxidative stress, damaging sperm DNA integrity and inducing sperm apoptosis. DLAT may play an important function in spermatogenic disorders through its role in cell death. Knocked down DLAT can downregulate LC3-II/Beclin-1 and thus inhibit autophagy [76], which can removal abnormal cell structures and misfolded proteins in spermatogenesis, if DLAT is expressed abnormally, it may lead to autophagy defects and affect cellular homeostasis in spermatogenesis [77]. The high expression of DLAT is closely related to cell proliferation, migration, and regulation of immune microenvironment[78]. DLAT positively correlates with Treg and Th2 cell infiltration [79], and its role in immunomodulation may affect the testicular immune microenvironment, which is dependent on an immunosuppressive environment for immune privilege in the testis, and DLAT abnormalities may disrupt this balance, inducing autoimmune testicular inflammation and further impairing spermatogenesis [80].

### Others

In other genes for cuproptosis, there may also be an association with spermatogenic disorders. Dihydrolipoamide branched-chain transacylase E2 (DBT) and its nanocomplexes can change mitochondrial membrane stability, reduce the release of cytochrome c, and inhibit apoptosis by adjusting the Bcl-2/Bax ratio [81]. In spermatogenic cells, DBT may regulate apoptosis through this mechanism. ATP7A and ATP7B are highly homologous P-type copper transporters, which regulate copper homeostasis through precise cell localization and transport mechanisms [82], copper ions are required for spermatogenesis, and may cause spermatogenesis disorders when ATP7A and ATP7B function abnormally. Glycine cleavage system protein H (GCSH) can inhibit the activation of the JAK-STAT signaling pathway [83], which is important in the self-renewal function of spermatogonial stem cells, leading to abnormal spermatogenesis.

## Conclusions and prospects

Cuproptosis is a novel mechanism of cell death, and the exploration of cuproptosis at the level of spermatogenic cells is helpful for further understanding the mechanisms of spermatogenic cell death. According to the current research evidence, cuproptosis is involved in the death process of spermatogenic cells, but its specific regulatory mechanism still needs further research. Meanwhile, cuproptosis may be interrelated with a variety of other types of spermatogenic death, and the study of the crosstalk between cuproptosis and other types of cell death and their regulatory mechanisms at the level of spermatogenic cells is conducive to further improving the molecular mechanism of spermatogenic cell death, enriching the pathological basis of male infertility, and providing a basis and ideas for the next step of developing new drugs or preparations to protect male fertility.
